# Revealing the mechanism of 755-nm long-pulsed alexandrite laser in inhibiting infantile hemangioma endothelial cells through transcriptome sequencing

**DOI:** 10.1007/s10103-023-03967-z

**Published:** 2024-01-18

**Authors:** Chen Ke, Changhan Chen, Ming Yang, Hao Chen, Liqun Li, Youhui Ke

**Affiliations:** 1 Department of Cosmetology, Wenzhou Hospital of Integrated Traditional Chinese and Western Medicine, Wenzhou, 325000 Zhejiang China; 2 Plastic Surgery, The First Affiliated Hospital of Wenzhou Medical University, Nanbaixiang, Wenzhou, 325000 Zhejiang China; 3 Wenzhou Key Laboratory of Laser Cosmetology, Wenzhou, 325000 Zhejiang China

**Keywords:** GSTM5, HemECs, Infantile hemangiomas, p62-Nrf2 pathway, Transcriptome sequencing

## Abstract

Laser therapy has shown promising outcomes in treating infantile hemangiomas. However, the molecular mechanisms underlying laser treatment for IH remain incompletely elucidated. This study aimed to unravel the molecular mechanisms of laser therapy in IH treatment. We evaluated the inhibitory effects of laser treatment on the proliferation and promotion of apoptosis in human hemangioma endothelial cells (HemECs) through cell counting kit-8 (CCK-8) assay, Hoechst 33342 staining, and flow cytometric analysis. Transcriptome sequencing analysis of HemECs following laser treatment revealed a significant decrease in the expression level of the GSTM5 gene. The qRT-PCR and western blot analysis also showed that GSTM5 expression in HemECs was downregulated compared to human umbilical vein endothelial cells (HUVECs), and concomitantly, the p62-Nrf2 pathway was suppressed. Using siRNA to downregulate GSTM5 expression, we observed that inhibiting GSTM5 expression could restrain cell proliferation, elevate intracellular ROS levels, and induce apoptosis in HemECs. Furthermore, upon inhibition of the p62-Nrf2 pathway using p62-specific siRNA, a significant decrease in GSTM5 expression and an elevation in intracellular ROS levels were noted in laser-treated HemECs. These findings suggested that laser treatment may operate by inhibiting the p62-Nrf2 pathway, thereby downregulating GSTM5 expression, elevating ROS levels, and consequently inducing apoptosis in HemECs.

**Supplementary Information:**

The online version contains supplementary material available at 10.1007/s10103-023-03967-z.

## Introduction

Infantile hemangioma (IH) is the most common benign tumor of infancy, typically appearing within 2 weeks of birth and subsequently undergoing a slow, spontaneous regression and tumor involution [1]. IH commonly manifests on the head, neck, and trunk, but can also occur in various anatomical locations such as the limbs, spine, and internal organs [2]. IH may sometimes be accompanied by complications such as ulceration, scarring, permanent deformities, organ dysfunction, and in certain cases; it can even lead to fatality [3, 4]. A noteworthy feature of proliferative hemangiomas is the accumulation of immature endothelial cells, and the endothelial cells derived from precursor cells constitute a significant element of IH [5, 6]. Research indicates that the primary histopathological feature of HemEC tissues is the abnormal proliferation of endothelial cells and rapid vascular growth [5, 7]. However, the molecular mechanisms responsible for driving this phenomenon remain incompletely understood. Therefore, delving into the underlying molecular mechanisms will contribute to a more comprehensive understanding of the etiology of IH.

The primary treatment modalities for IH currently include the use of beta-blockers, surgical intervention, and laser treatment. These treatments offer precise targeting of the affected area, reducing the impact on surrounding normal tissue and helping to minimize trauma and inflammatory responses. However, patients should remain vigilant during the later stages of treatment, as some therapeutic approaches may induce fibroblast generation, potentially increasing the risk of scar formation [8, 9]. Among these treatment modalities, beta-blockers are the first-line therapy for IH currently. Topical application of beta-blockers can be employed in the treatment of superficial and thin IH, but their efficacy is limited for some thicker IH lesions. Moreover, residual hemangioma following beta-blocker treatment can result in cosmetic defects [10]. Surgical interventions are presently used sparingly due to their propensity for scarring and relatively higher recurrence rates [11]. Laser therapy predominantly encompasses pulsed dye laser (PDL) (wavelengths of 585 nm or 595 nm), Nd:YAG (wavelength 1064 nm), and the long-pulsed alexandrite laser (wavelength 755 nm). PDL’s limited tissue penetration for thicker IH yields suboptimal outcomes. In contrast, Nd:YAG’s narrow treatment window complicates precise control, potentially damaging deep dermal tissues and causing scarring. Hence, neither PDL nor Nd:YAG are deemed optimal for treating thicker IH cases [12]. Recent research revealed that the 755-nm long-pulsed alexandrite laser boasts nearly twice the tissue penetration compared to the PDL laser. Moreover, it has been established as a secure and effective modality for addressing deeper vascular malformations [13]. The presently accepted mechanism of laser therapy for IH is founded on the selective photothermal effect targeting oxygenated hemoglobin, leading to the rupture of oxygenated red blood cells, vessel occlusion, degeneration, and necrosis of endothelial cells. This sequence of events disrupts the capillaries within the vascular malformation, culminating in its contraction [14]. Nevertheless, a comprehensive understanding of the precise molecular mechanisms underpinning these effects necessitates further exploration. Therefore, this study aims to investigate the effects of laser treatment on hemangioma endothelial cells (HemECs). Subsequently, RNA transcriptome sequencing was conducted on HemEC cells before and after laser treatment to delve into the molecular mechanisms underlying laser therapy for IH.

## Materials and methods

### Study design

This study constitutes an in vitro experimental investigation focusing on laser treatment and assessment. Initially, laser effects on the proliferation and apoptosis of HemECs were observed through cellular experiments. Subsequently, RNA transcriptome sequencing was conducted on both non-laser-exposed and laser-exposed HemEC cells, aiming to elucidate the genetic alterations in HemECs following laser irradiation. Furthermore, through additional cellular experiments, a comprehensive exploration of potential impact on signaling pathways and the modulation of intracellular reactive oxygen species (ROS) levels post laser exposure on HemECs was conducted. All experiments were replicated three times to ensure robustness and reliability.

### Laser setup

**Fig. 1:**
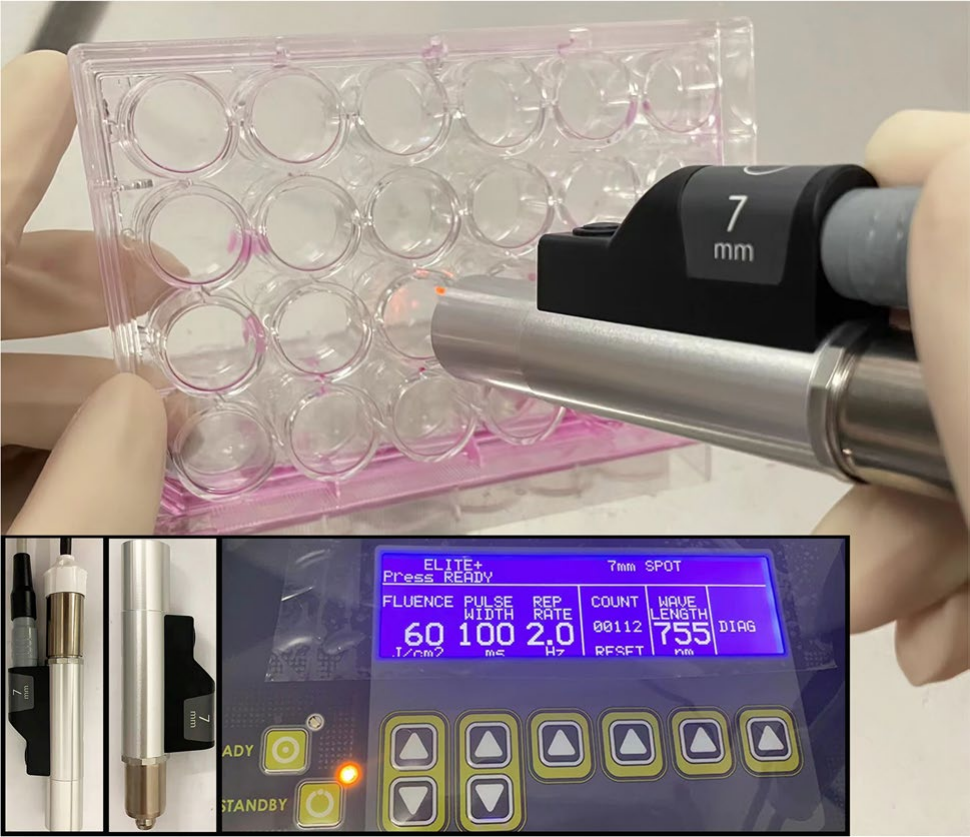
Experimental procedure for laser irradiation on HemECs

The HemECs were divided into the control group (HemECs group) and laser group (Laser group), with the laser group undergoing treatment using a 755-nm long-pulsed alexandrite laser (Cynosure, LLC., Westford, USA), as shown in Fig. 1. The laser treatment parameters included a wavelength of 755 nm, pulse width of 100 ms, energy density of 60 J/cm^2^, and an 7-mm beam diameter.

### Cell culture

Human umbilical vein endothelial cells (HUVECs) and HemECs were both obtained from iCell Bioscience Inc (Shanghai, China). HUVECs and HemECs were cultured in high-glucose DMEM medium (Procell, Wuhan, China) supplemented with 10% fetal bovine serum (Procell, Wuhan, China) and 1% penicillin–streptomycin-gentamicin solution (Solarbio, Beijing, China). All cells were cultivated in a humid environment with 5% CO_2_ at a temperature of 37 °C.

### Cell Counting Kit-8 (CCK-8) assay

Cell proliferation ability was determined using the CCK-8 assay kit (BBI Life Sciences, Shanghai, China), strictly following the manufacturer’s instructions. Treated HemECs were seeded in a 96-well plate at a density of 2000 cells per well. Subsequently, 10 µL of CCK-8 reagent was added to each well, followed by incubation at 37 °C in the absence of light for 1 h. Subsequently, absorbance was measured at a wavelength of 450 nm.

### Hoechst 33,342 staining

Hoechst 33,342 staining (Beyotime Biotechnology, Shanghai, China) was employed to assess changes in cellular morphology associated with cell death. Treated cell groups were seeded into 6-well plates and cultured in DMEM/F12 medium for 24 h. Following removal of the culture medium, cells were co-incubated with Hoechst 33,342 for 30 min, followed by PBS washing. Following that, apoptotic conditions within the diverse cell groups were examined utilizing an fluorescence microscope.

### Flow cytometric analysis

In this study, flow cytometric analysis was employed to assess cellular apoptosis. A mixture containing 100 µL of cells at a density of 1 × 10^5^ cells and binding buffer was added to a 5-mL culture tube. Following the manufacturer’s instructions, dual staining with FITC Annexin V and propidium iodide was performed using the FITC Annexin V Apoptosis Detection Kit (BD Bioscience, East Rutherford, NJ, USA). Cellular analysis was conducted using a flow cytometer (FACScan, BD Biosciences, East Lutherford, USA).

### Transcriptome sequencing

After conducting Illumina transcriptome sequencing (Novogene, Beijing, China) on the HemEC group and the Lsaer group, differential gene expression (DEG) was performed using the DESeq2 1.16.1 software. The criteria for selecting DEGs were set as *P* < 0.05 and |log2FoldChange|> 0.5. The results of the DEG selection were presented in the form of clustered heatmaps and volcano plots [15].

### Quantitative Real-Time Polymerase Chain Reaction (qRT-PCR)

RNA extraction from cells was carried out using TRIzol reagent (Invitrogen, San Francisco, CA, USA). Reverse transcription of cDNA and real-time quantitative PCR were performed following the instructions provided with the reverse transcription kit, and real-time fluorescent PCR kit. GAPDH was employed as an internal reference. The relative expression levels of genes in the cells were calculated using the 2^−ΔΔCT^ method for relative quantification, with the primer sequences listed in Table 1.

**Table 1:** The primer sequence

Gene name	Forward	Reverse
GSTM5	5′-TGGACTTTCCCAATCTGCCC-3′	5′-ATGTAGCGCAGGATGGCATT-3′
P62	5′-AATGGCACCTTCCTGACGC-3′	5′-AGGAACTCCCGCTGGTAAAC-3′
Nrf2	5′-CTTCTAGTTCGGACGCGGTG-3	5′-ATCCATGTCCTGTCCCTTGG-3′
GAPDH	5′-TCAGCCGCATCTTCTTTTGC-3′	5′-CCCAATACGACCAAATCCGT-3′

### Western blot analysis

Protein extraction from processed cells was followed by quantification of protein concentration using the BCA Protein Assay Kit (Beyotime, Shanghai, China). Protein samples were separated on 10% sodium dodecyl sulfate–polyacrylamide gel electrophoresis (SDS-PAGE) gels, and subsequently transferred onto polyvinylidene fluoride (PVDF) membranes. The membranes were then blocked with TBST containing 5% skim milk for 30 min. The PVDF membranes were probed with monoclonal anti-GSTM5 antibody (1:5000, Proteintech, Wuhan, China), monoclonal anti-P62 antibody (1:5000, Proteintech, Wuhan, China), monoclonal anti-Nrf2 antibody (1:1000, Abcam, Cambridge, UK), and monoclonal anti-GAPDH antibody (1:50,000, Proteintech, Wuhan, China) at 4°C overnight. Following this, the PVDF membranes were incubated with corresponding secondary antibodies at room temperature for 30 min and subsequently subjected to chemiluminescence imaging and analysis using a chemiluminescence imaging system. The band intensities were quantified using the Image J software.

### Small Interfering RNA (siRNA) transfection

Silencing of glutathione S-transferase Mu 5 (GSTM5) and p62 was achieved by transfecting specific siRNA (GenePharma, Shanghai, China) into HemECs using Lipofectamine 2000 (Invitrogen, San Francisco, CA, USA), following the manufacturer’s instructions. Following the completion of transfection, the cells were cultured for subsequent experiments.

### Colony formation assay

After successful cell transfection, cells were subjected to digestion using trypsin (Thermo Fisher Scientific Gibco, Carlsbad, CA, USA) and prepared into a cell suspension. The cell suspension was seeded into a 6-well plate, with 2000 cells per well. The cells were then cultured under conditions of 37 °C and 5% CO_2_ for a duration of 2 weeks. Following this period, the cells were fixed with 4% paraformaldehyde for 10 min, stained with 0.1% crystal violet solution (Solarbio, Beijing, China), and bacterial colonies were counted under an optical microscope.

### Intracellular ROS detection

Following various treatments, HemEC cells were incubated with the ROS fluorescent probe DCFH-DA at 37 °C for 20 min. Subsequently, observations were made using fluorescence microscopy, and the ROS levels were quantified using flow cytometry.

### Statistical analyses

All data are presented as mean ± standard deviation (SD) and were statistically analyzed and graphed using the GraphPad Prism 9.0. Two-group comparisons were performed using the independent *t*-test, with *P* < 0.05 considered statistically significant. All experiments were conducted in triplicate.

## Results

### Laser treatment suppressed HemEC proliferation and promoted HemEC apoptosis

**Fig. 2:**
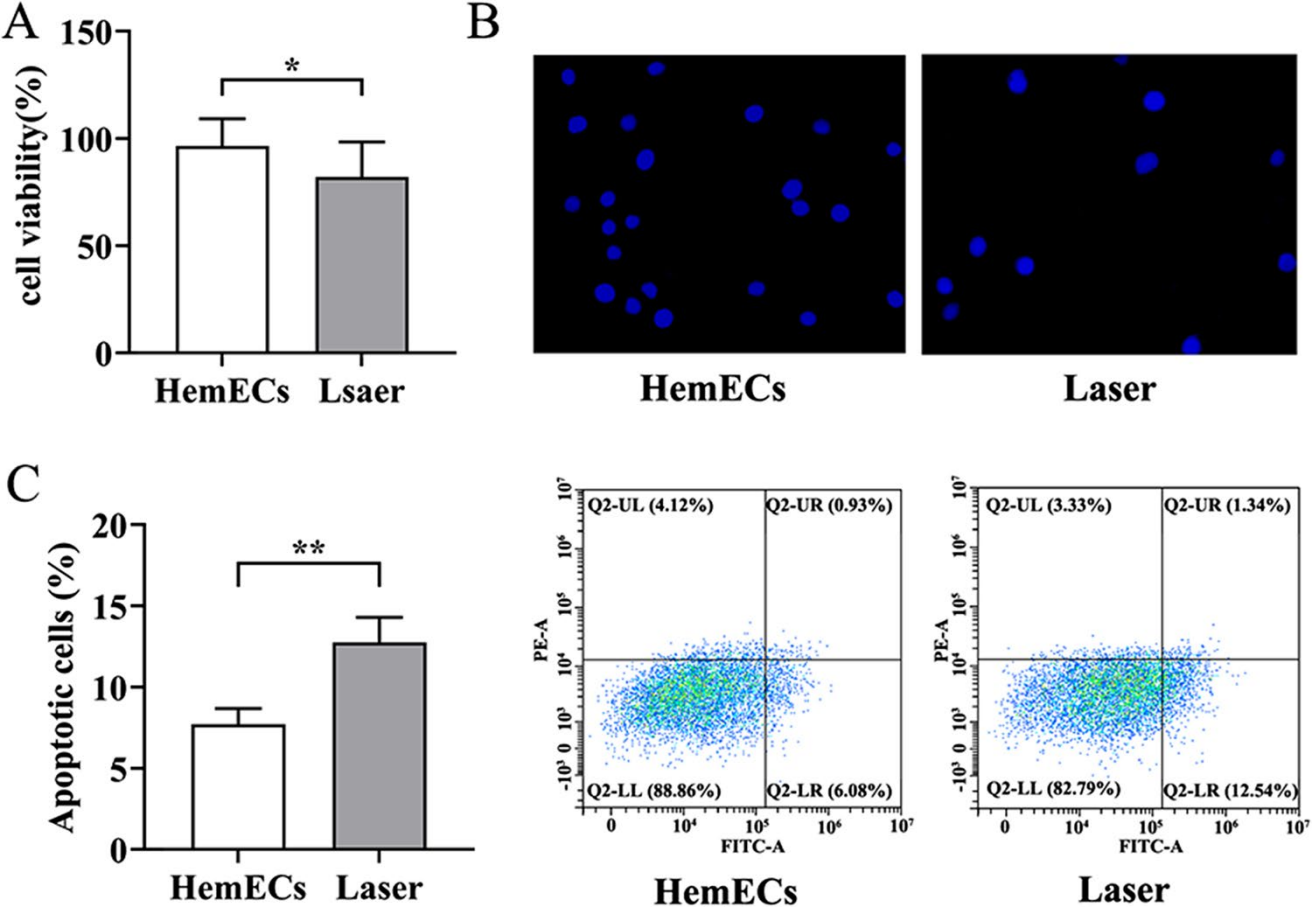
Laser treatment could inhibit the proliferation of HemEC cells and promoted apoptosis in HemEC cells. **A**, Results of the CCK-8 experiment. **B** Hochest 33,342 staining. **C** Flow cytometry analysis for cell apoptosis. *P < 0.05, **P < 0.01

CCK-8 results revealed a significant decrease in cell proliferation capacity in the Laser group compared to the HemEC group (*P* < 0.05) (Fig. 2A). Additionally, in order to assess the impact of laser treatment on HemECs apoptosis, we conducted Hoechst 33,342 staining and flow cytometry analysis. The outcomes revealed a notable elevation in the apoptosis rate within the Laser group when contrasted with the HemEC group (Fig. 2B, C). These findings collectively indicated that laser treatment can inhibit HemEC cell proliferation and promote HemEC cell apoptosis.

### Laser treatment downregulated the expression level of GSTM5 in HemECs

**Fig. 3:**
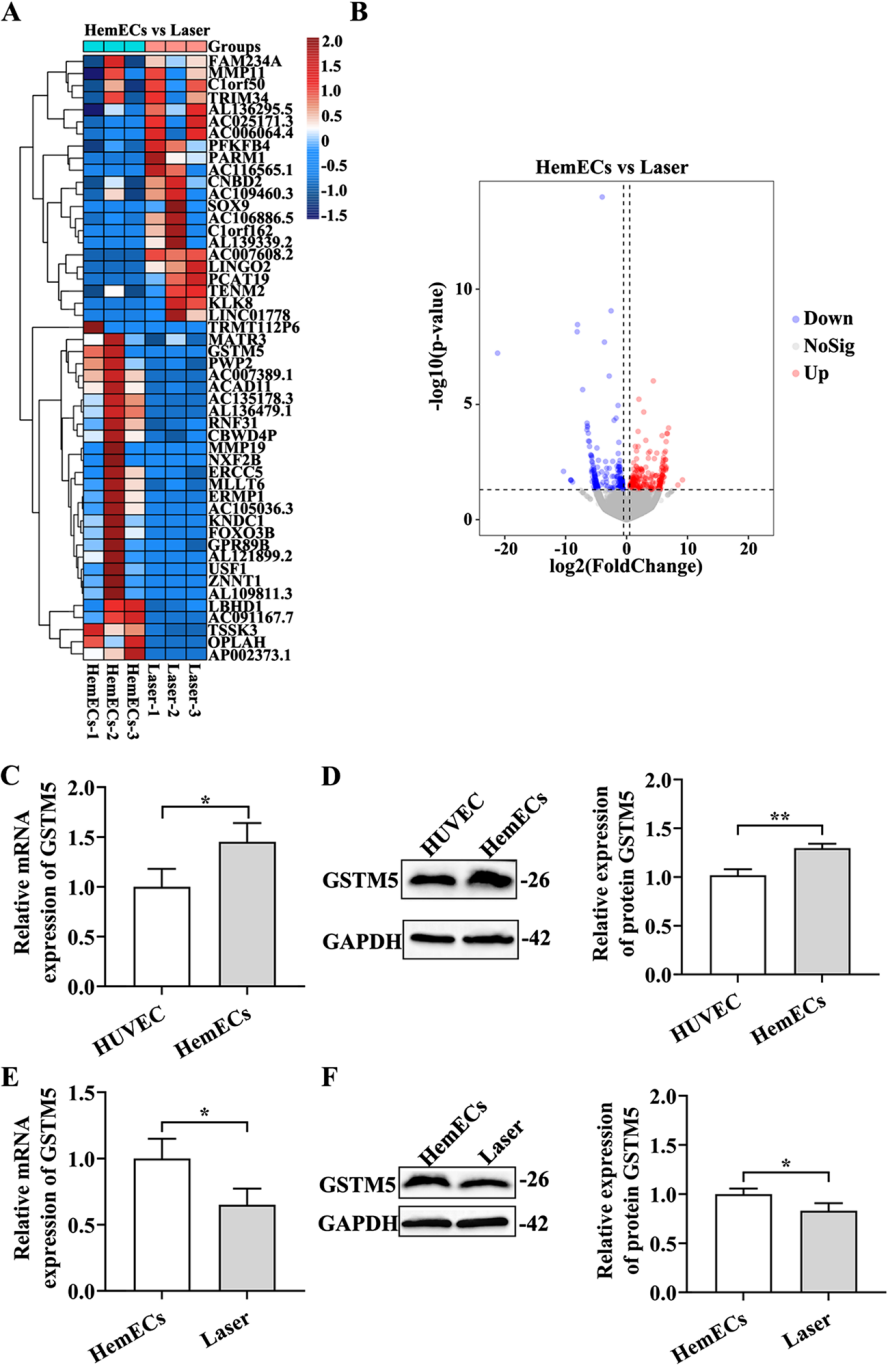
Laser-mediated controlled over GSTM5 and the regulation of other gene expressions in HemECs. **A** Hierarchical heatmap of differentially expressed genes. The heatmap was generated using log2(TPM + 1) values of the differentially expressed genes, with red and blue indicating high and low expression levels of the gene TPM, respectively. **B** Volcano plot of differentially expressed genes, where red represents upregulated genes and blue represents downregulated genes. **C** Quantitative analysis of GSTM5 mRNA expression in HemECs. **D** Western blot images and quantitative analysis of GSTM5 protein expression levels in HemECs. **E** Quantitative analysis of GSTM5 mRNA expression in HemECs following laser treatment. **F** Western blot images and quantitative analysis of GSTM5 protein expression levels in HemECs following laser treatment. *P < 0.05, **P < 0.01

In order to elucidate the molecular mechanisms underlying the effects of laser treatment on HemECs, we conducted RNA transcriptome sequencing on cells from both the HemEC group and the Laser group. Using the DESeq2 software, a total of 487 DEGs were identified (*P* < 0.05, |log2FoldChange|> 0.5), including 242 upregulated and 236 downregulated genes (Fig. 3A, B; Supplementary Table 1). Of particular note, there was a significant decrease in the expression of GSTM5 in HemECs following laser treatment.

Transcriptomic results revealed a significant decrease in GSTM5 expression in HemECs after laser treatment. To ascertain the expression pattern of GSTM5 in HemECs and its modulation following laser treatment, qRT-PCR and western blot analyses were conducted. The qRT-PCR and western blot analysis findings indicated a significant upregulation of GSTM5 expression in HemECs compared to human umbilical vein endothelial cells (HUVECs) (*P* < 0.05) (Fig. 3C, D), and a pronounced downregulation after laser treatment (*P* < 0.05) (Fig. 3E, F). These results underscored the capacity of laser treatment to downregulate the expression of the GSTM5 gene in HemECs.

### Laser-induced inhibition of GSTM5 expression via the suppression of the p62-Nrf2 pathway led to the inhibition of HemEC proliferation and the promotion of HemEC apoptosis

**Fig. 4:**
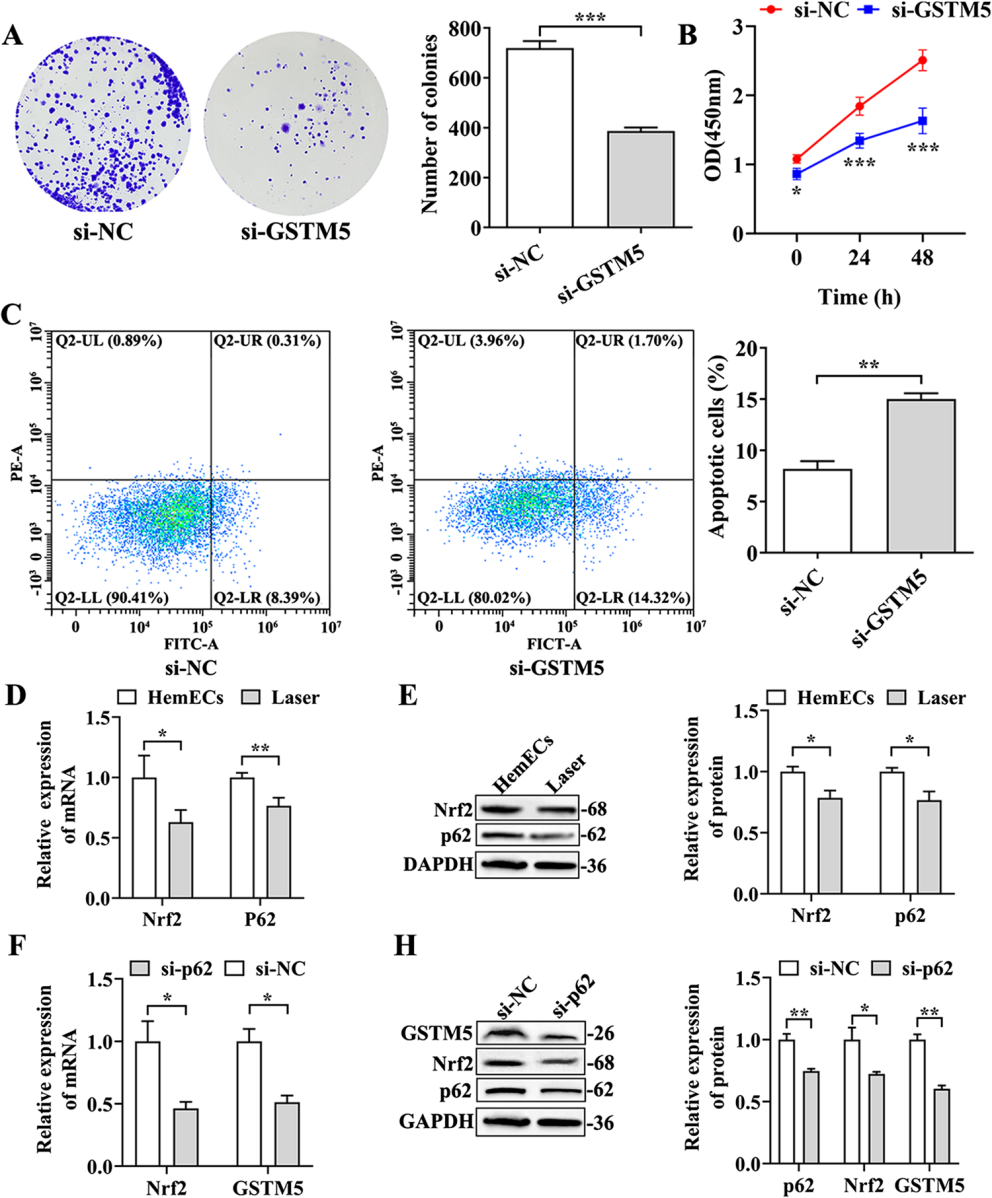
Laser treatment of HemEC cells could inhibit the p62-Nrf2 pathway, downregulated GSTM5 expression, inhibited HemECs proliferation, and promoted HemEC apoptosis. **A** Colony formation assay to assess cell proliferation capability. **B** CCK-8 assay to evaluate cell proliferation capability. **C** Flow cytometry assay to measure cell apoptosis. **D** Quantitative analysis of p62 and Nrf2 mRNA expression in HemECs. **E** Western blot images and quantitative analysis of p62 and Nrf2 protein expression levels in HemECs. **F** Quantitative analysis of Nrf2 and GSTM5 mRNA expression in HemECs following p62 silencing. **H** Western blot images and quantitative analysis of Nrf2, p62, and GSTM5 protein expression levels in HemECs following p62 silencing. *P < 0.05, **P < 0.01, ***P < 0.001

To investigate the impact of GSTM5 on HemEC proliferation and apoptosis, we conducted transfections with specific siRNAs targeting and silencing GSTM5 mRNA, followed by colony formation assay, CCK-8, and flow cytometry assays. Colony formation assay results demonstrated a significant reduction in cell count in the si-GSTM5 group compared to the si-NC group (Fig. 4A). The CCK-8 assay results exhibited a significant reduction in cell proliferation capacity in the si-GSTM5 group as compared to the si-NC group (*P* < 0.001) (Fig. 4B). Flow cytometry assay findings exhibited a notable elevation in the apoptosis rate within the si-GSTM5 group when contrasted with the si-NC group (*P* < 0.01) (Fig. 4C). These experimental outcomes collectively indicated that silencing GSTM5 inhibited HemEC proliferation and promoted apoptosis.

To investigate changes in the p62-Nrf2 pathway following laser treatment of HemECs, we conducted qRT-PCR and western blot analyses to assess the regulatory effect of laser treatment on the p62-Nrf2 pathway. Results from qRT-PCR and Western blot analyses indicated that the p62-Nrf2 pathway was inhibited following laser treatment of HemECs (Fig. 4D, E), suggesting the capability of laser treatment to suppress the p62-Nrf2 pathway. To further explore the impact of the p62-Nrf2 pathway on GSTM5, we designed specific siRNAs to target and silence p62 mRNA, leading to inhibition of the p62-Nrf2 pathway. The efficiency of p62-Nrf2 pathway inhibition was validated through qRT-PCR and western blot analyses. confirming the successful suppression of the p62-Nrf2 pathway. Moreover, upon inhibition of the p62-Nrf2 pathway, a significant decrease in GSTM5 expression was observed (*P* < 0.05) (Fig. 4F, H). These findings collectively indicated that laser treatment of HemEC cells can suppress the p62-Nrf2 pathway, thereby downregulating GSTM5 expression.

### Laser treatment enhanced intracellular ROS levels in HemECs

**Fig. 5:**
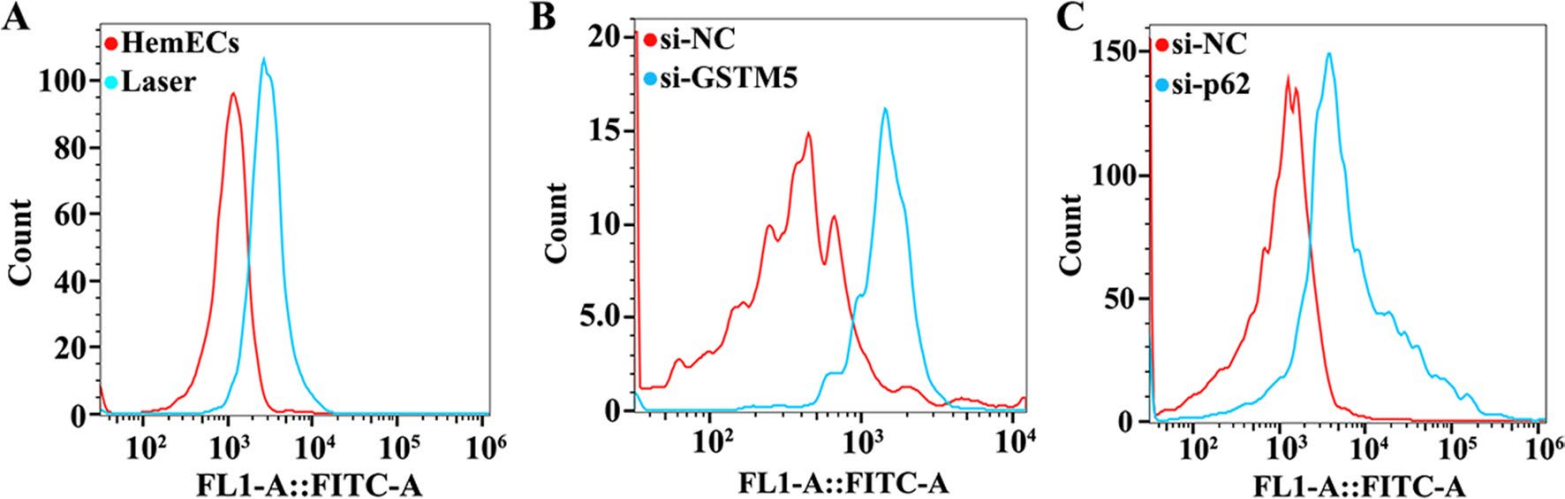
Elevated levels of intracellular ROS in HemECs. **A** Laser treatment upregulates intracellular ROS levels in HemECs. **B** Silencing GSTM5 upregulates intracellular ROS levels in HemECs. **C** Inhibition of the p62-Nrf2 pathway upregulates intracellular ROS levels in HemECs

To investigate the impact of silencing GSTM5 and the p62-Nrf2 pathway on intracellular ROS in HemEC cells, we measured ROS levels using flow cytometry assays. Experimental results revealed that laser treatment, GSTM5 silencing, and inhibition of the p62-Nrf2 pathway all enhanced intracellular ROS levels in HemECs (Fig. 5). Taken together with the previous research findings, laser treatment of HemECs could suppress the p62-Nrf2 pathway, downregulate GSTM5 expression, and consequently elevate ROS levels in HemECs cells.

## Discussion

Propranolol is currently considered the first-line treatment approach for IH, demonstrating high therapeutic efficacy with minimal side effects, and multiple studies have confirmed its effectiveness and safety [16, 17]. However, even with systemic treatment using propranolol, many IH cases only exhibit partial regression, leaving residual vascular anomalies that can lead to cosmetic concerns. Furthermore, some patients may experience side effects such as hypoglycemia, hypotension, and bradycardia [13]. Thus, in such scenarios, laser therapy presents a viable alternative, and laser has demonstrated promising outcomes in treating IH [18]. However, the molecular mechanisms underlying laser therapy for IH remain incompletely elucidated. To delve into the molecular basis of laser therapy for IH, this study conducted transcriptomic analysis on untreated and laser-treated HemECs. The findings revealed a significant downregulation of the GSTM5 gene expression in HemECs following laser treatment. GSTM5, an antioxidant gene, has been implicated in the progression of solid tumors such as colorectal cancer [19], bladder cancer [20], and lung adenocarcinoma [21]. Nevertheless, the role of GSTM5 in IH remains unclear. The study’s results indicated GSTM5 is overexpressed in HemECs. Further in vitro experimental data suggested that laser treatment may downregulate GSTM5 expression by inhibiting the p62-Nrf2 pathway, triggering oxidative damage, and consequently promoting apoptosis in HemECs.

Glutathione-S-transferases (GSTs), discovered in the liver in 1961, comprise a versatile group of enzymes involved in catalyzing oxidation–reduction reactions [22]. GST comprises a multifunctional group of enzymes catalyzing various redox reactions. These enzymes collaborate with other antioxidants to eliminate reactive oxygen species, thereby safeguarding cells from oxidative damage [23]. Moreover, they are believed to participate in detoxification processes, converting potential carcinogenic metabolites into inactive forms. Across various cell types, GSTs exhibit notably elevated expression levels in aggressive cancers, suggesting their crucial involvement in tumor progression [24–26]. GSTM5, located on human chromosome 1, stands as a member within the GSTs gene family [27]. Among them, cytosolic GSTs like GSTM5 are deeply involved in glutathione (GSH) metabolism, engage in protein interactions with key kinases regulating apoptosis and proliferation, thus modulating cellular signaling [28]. Existing evidence has indicated its involvement in the development of various cancers, including gastric cancer [29] and urothelial carcinoma [30]. However, the exact mechanistic role of GSTM5 in cancer remains elusive. Research has revealed that GSTM5 is upregulated in tumors such as Barrett’s adenocarcinoma [31], lung adenocarcinoma [21], bladder cancer [20], prostate cancer, and breast cancer [28]. Conversely, it is upregulated in colorectal cancer [19]. Additionally, GSTM5 has been closely associated with processes including angiogenesis, cell proliferation, cell migration, and apoptosis [29]. Consistent with these findings, our research demonstrate significant upregulation of GSTM5 protein and mRNA expression levels in HemECs, aligning with observations made by Liu et al. in colorectal cancer [19]. Furthermore, following laser treatment of HemECs, both GSTM5 protein and mRNA expression levels exhibit a declining trend. This, coupled with the transcriptomic analysis results, indicated that laser treatment can downregulate GSTM5 expression in HemECs. As a GST member, GSTM5 is believed to interact with various kinases, engaging in processes of apoptosis and cell proliferation [32]. The experimental outcomes from this study underscored that both laser treatment and GSTM5 silencing can inhibit cell proliferation and promote apoptosis in HemECs. These findings underscored the capacity of laser treatment to downregulate GSTM5 expression, thereby restraining cell proliferation and fostering apoptosis.

Nuclear factor erythroid 2-related factor 2 (Nrf2) is a critical regulatory factor in cellular responses to oxidative stress, inflammation, metabolic stress, and phototoxicity [33]. Overactivation of Nrf2 can minimize oxidative stress induced by tumors, thereby reducing tumor cell apoptosis and promoting tumor development [34]. In cells, the accumulation of Nrf2 is achieved through competitive binding between p62 and Keap1. This process effectively prevents Nrf2 from binding to Keap1, leading to Nrf2 gradual accumulation within cells, subsequently promoting tumorigenesis [35]. Simultaneously, studies have observed elevated levels of p62 expression in various tumors. Overexpression of p62 can induce cell proliferation and invasion [36], and it plays a role as an oncogene in tumor formation and progression by modulating Nrf2 [37]. Thus, the activation of the p62-Nrf2 pathway holds significant importance in the development of tumors. Research by Li et al. [38] has confirmed that p62 activates the Nrf2 signal by sequestering Keap1, resulting in the upregulation of GSTM5 gene expression, suggesting potential regulation of GSTM5 by the p62-Nrf2 pathway. Consistent with existing research, in our study, we observed that the p62-Nrf2 pathway in HemECs was suppressed after laser treatment. Moreover, following inhibition of the p62-Nrf2 pathway, GSTM5 expression was downregulated. Our findings suggested that laser treatment of HemECs may downregulate GSTM5 expression by inhibiting the p62-Nrf2 pathway, consequently restraining cell proliferation and promoting apoptosis.

This study also observed an elevation in intracellular ROS levels. ROS is typically generated during the metabolic processes of aerobic cells and can regulate various physiological functions such as transcription factor activation, gene expression, and cell proliferation [39]. In this study, the increase in ROS levels within the HemECs could potentially be attributed to the inhibition of the p62-Nrf2 pathway and the downregulation of GSTM5 gene expression following laser irradiation. p62 is an autophagy adaptor protein that promotes the degradation of misfolded proteins through autophagolysosomes and the ubiquitin–proteasome pathway [40]. Activation of p62 can enhance autophagy-mediated Keap1 degradation, thereby releasing Nrf2 and inducing its nuclear translocation [41]. Under conditions of cellular oxidative stress, Nrf2 translocates to the nucleus, where it binds to antioxidant response elements (AREs) and activates the expression of cellular protective and antioxidant enzymes, suppressing ROS production [42]. Furthermore, in an acute kidney injury model, Liao et al. [43] found that p62 knockdown significantly reduced Nrf2 protein expression, accompanied by increased oxidative stress. Consequently, in this study, inhibiting p62 resulted in the prevention of Nrf2 translocation to the nucleus, leading to ROS accumulation. Simultaneously, as a member of the GSTs family, GSTM5 serves as an antioxidative gene [44], participating in intracellular glutathione (GSH) metabolic reactions to reduce levels of reactive oxygen species (ROS) [20, 45]. In this study, the decreased expression of GSTM5 following laser treatment of HemEC cells and GSTM5 knockdown disrupted the GSTs/GSH balance [45], consequently elevating ROS levels within HemECs. However, excessive ROS can disrupt the cellular redox balance and lead to cell death [46]. Maintaining redox balance is crucial for both normal and cancer cells. When cellular redox balance is disrupted, such as when the levels of reactive oxygen species exceed intrinsic antioxidant capacity, cells may undergo cell death [20]. Recent research on the biological functions of ROS has revealed that elevated ROS levels can activate multiple cell death pathways, thereby limiting tumor development [34]. Additionally, inhibiting both GSH and ROS accumulation can disrupt intracellular redox homeostasis, further amplifying oxidative stress and affecting cell viability [45].

In summary, we speculate that laser treatment of HemECs may increase ROS levels through the inhibition of the p62-Nrf2 pathway and the downregulation of GSTM5 expression, consequently suppressing cell proliferation and promoting apoptosis.

## Conclusion

In conclusion, our study findings indicated that laser treatment can downregulate GSTM5 expression by inhibiting the p62-Nrf2 pathway, leading to an elevation in intracellular ROS levels and ultimately promoting apoptosis in HemECs. This investigation delves into the roles of GSTM5 and the p62-Nrf2 pathway in the pathogenesis of IH, laying the foundation for a deeper understanding and intervention strategies to enhance the effectiveness of laser therapy for IH. Moreover, it provides a scientific basis for the development of more precise and efficient treatment approaches, offering theoretical support for the clinical application of laser therapy in IH. In the future, we will conduct extensive in vivo studies to comprehensively assess the efficacy and safety of laser treatment in a more realistic environment.

## Supplementary Information

Below is the link to the electronic supplementary material.

Supplementary file1 (XLSX 34 KB)

## References

[1] Al-Haddad C, El Salloukh NA, El Moussawi Z (2019) beta-blockers in the treatment of periocular infantile hemangioma. Curr Opin Ophthalmol 30(5):319–325. 10.1097/ICU.0000000000000591 31394556 10.1097/ICU.0000000000000591

[2] Chinnadurai S, Sathe NA, Surawicz T (2016) Laser treatment of infantile hemangioma: A systematic review. Lasers Surg Med 48(3):221–233. 10.1002/lsm.22455 26711436 10.1002/lsm.22455

[3] Leung AKC, Lam JM, Leong KF, Hon KL (2021) Infantile hemangioma: An updated review. Curr Pediatr Rev 17(1):55–69. 10.2174/1573396316666200508100038 32384034 10.2174/1573396316666200508100038

[4] Nazemian S, Sharif S, Childers ELB (2023) Infantile hemangioma: A common lesion in a vulnerable population. Int J Environ Res Public Health 20(8). 10.3390/ijerph20085585 10.3390/ijerph20085585 PMC10139075 37107867

[5] Zhao Y, Li D, Han Y, Wang H, Du R, Yan Z (2022) The ester derivatives obtained by C-ring modification of podophyllotoxin-induced apoptosis and inhibited proliferation in hemangioma endothelial cells via downregulation of PI3K/Akt signaling pathway. Chem Biol Drug Des 99(6):828–838. 10.1111/cbdd.14034 35184389 10.1111/cbdd.14034

[6] Sun Y, Qiu F, Hu C, Guo Y, Lei S (2022) Hemangioma endothelial cells and hemangioma stem cells in infantile hemangioma. Ann Plast Surg 88(2):244–249. 10.1097/SAP.0000000000002835 35023872 10.1097/SAP.0000000000002835

[7] Jin W, Chen L, Gao F, Yang M, Liu Y, Wang B (2020) Down-regulation of miR-556–3p inhibits hemangioma cell proliferation and promotes apoptosis by targeting VEGFC. Cell Mol Biol (Noisy-le-grand) 66(5):204–207 33040837

[8] Jiang JC, Xu Q, Fang S, Gao Y, Jin WW (2021) Sequelae after involution of superficial infantile hemangioma: Early intervention with 595-nm pulsed laser combined with 755-nm long-pulsed alexandrite laser versus Wait-and-See. Clin Cosmet Investig Dermatol 14:37–43. 10.2147/CCID.S279140 33469332 10.2147/CCID.S279140 PMC7811447

[9] Hu H, Song P, Yang J, Wang X, Chen Z, Fang J (2020) Therapeutic effect of high-frequency ultrasound-assisted dye laser on hemangioma and its influence on serum HIF-1alpha in patients. J Clin Lab Anal 34(1):e22970. 10.1002/jcla.22970 31568612 10.1002/jcla.22970 PMC6977139

[10] Couto JA, Greene AK (2014) Management of problematic infantile hemangioma using intralesional triamcinolone: Efficacy and safety in 100 infants. J Plast Reconstr Aesthet Surg 67(11):1469–1474. 10.1016/j.bjps.2014.07.009 25104131 10.1016/j.bjps.2014.07.009

[11] Qiu Y, Lin X, Ma G, Chang L, Jin Y, Chen H, Hu X (2015) Eighteen cases of soft tissue atrophy after intralesional bleomycin a5 injections for the treatment of infantile hemangiomas: A long-term follow-up. Pediatr Dermatol 32(2):188–191. 10.1111/pde.12422 25640925 10.1111/pde.12422

[12] Wu J, Zhou F, Gao Y (2021) Efficacy evaluation of 755-nm long-pulse alexandrite laser combined with 0.5% Timolol Maleate Eye Drops in the treatment of thicker infantile hemangioma. Clin Cosmet Investig Dermatol 14:1621–1628. 10.2147/CCID.S330411 34785921 10.2147/CCID.S330411 PMC8590841

[13] Ziad K, Badi J, Roaa Z, Emily AH (2023) Laser treatment of infantile hemangioma. J Cosmet Dermatol 22 Suppl 2:1–7. 10.1111/jocd.15671 10.1111/jocd.15671 36774645

[14] He HY, Shi WK, Jiang JC, Gao Y, Xue XM (2022) An exploration of optimal time and safety of 595-nm pulsed dye laser for the treatment of early superficial infantile hemangioma. Dermatol Ther 35(5):e15406. 10.1111/dth.15406 35199898 10.1111/dth.15406 PMC9285537

[15] Zhao Y, Li X, Zhang H, Yan M, Jia M, Zhou Q (2022) A transcriptome sequencing study on genome-wide gene expression differences of lung cancer cells modulated by fucoidan. Front Bioeng Biotechnol 10:844924. 10.3389/fbioe.2022.844924 35299642 10.3389/fbioe.2022.844924 PMC8923512

[16] Kridin K, Pam N, Bergman R, Khamaysi Z (2020) Oral propranolol administration is effective for infantile hemangioma in late infancy: A retrospective cohort study. Dermatol Ther 33(3):e13331. 10.1111/dth.13331 32216160 10.1111/dth.13331

[17] Pam N, Kridin K, Khamaysi Z (2021) Propranolol for infantile hemangioma: Evaluating efficacy and predictors of response and rebound growth. Dermatol Ther 34(3):e14936. 10.1111/dth.14936 33704861 10.1111/dth.14936

[18] Baumgartner J, Simaljakova M, Babal P (2016) Extensive angiokeratoma circumscriptum — successful treatment with 595-nm variable-pulse pulsed dye laser and 755-nm long-pulse pulsed alexandrite laser. J Cosmet Laser Ther 18(3):134–137. 10.3109/14764172.2015.1114643 26736060 10.3109/14764172.2015.1114643

[19] Liu F, Xiao XL, Liu YJ, Xu RH, Zhou WJ, Xu HC, Zhao AG, Xu YX, Dang YQ, Ji G (2021) CircRNA_0084927 promotes colorectal cancer progression by regulating miRNA-20b-3p/glutathione S-transferase mu 5 axis. World J Gastroenterol 27(36):6064–6078. 10.3748/wjg.v27.i36.6064 34629820 10.3748/wjg.v27.i36.6064 PMC8476332

[20] Jou YC, Wang SC, Dia YC, Wang ST, Yu MH, Yang HY, Chen LC, Shen CH, Liu YW (2021) Anti-cancer effects and tumor marker role of glutathione S-transferase Mu 5 in human bladder cancer. Int J Mol Sci 22(6). 10.3390/ijms22063056 10.3390/ijms22063056 PMC8002531 33802702

[21] Hao X, Zhang J, Chen G, Cao W, Chen H, Chen S (2022) Aberrant expression of GSTM5 in lung adenocarcinoma is associated with DNA hypermethylation and poor prognosis. BMC Cancer 22(1):685. 10.1186/s12885-022-09711-0 35729618 10.1186/s12885-022-09711-0 PMC9214983

[22] Booth J, Boyland E, Sims P (1961) An enzyme from rat liver catalysing conjugations with glutathione. Biochem J 79(3):516–524. 10.1042/bj0790516 16748905 10.1042/bj0790516 PMC1205680

[23] Zivkovic M, Stankovic A, Djuric T, Koncar I, Kolakovic A, Djurdjevic V, Davidovic L, Alavantic D (2014) Effects of glutathione S-transferase T1 and M1 deletions on advanced carotid atherosclerosis, oxidative, lipid and inflammatory parameters. Mol Biol Rep 41(2):1157–1164. 10.1007/s11033-013-2962-z 24407598 10.1007/s11033-013-2962-z

[24] Louie SM, Grossman EA, Crawford LA, Ding L, Camarda R, Huffman TR, Miyamoto DK, Goga A, Weerapana E, Nomura DK (2016) GSTP1 is a driver of triple-negative breast cancer cell metabolism and pathogenicity. Cell Chem Biol 23(5):567–578. 10.1016/j.chembiol.2016.03.017 27185638 10.1016/j.chembiol.2016.03.017 PMC4876719

[25] Singh RR, Reindl KM (2021) Glutathione S-transferases in cancer. Antioxidants (Basel) 10(5). 10.3390/antiox10050701 10.3390/antiox10050701 PMC8146591 33946704

[26] Kearns PR, Chrzanowska-Lightowlers ZM, Pieters R, Veerman A, Hall AG (2003) Mu class glutathione S-transferase mRNA isoform expression in acute lymphoblastic leukaemia. Br J Haematol 120(1):80–88. 10.1046/j.1365-2141.2003.04039.x 12492580 10.1046/j.1365-2141.2003.04039.x

[27] Alexander M, Karmaus W, Holloway JW, Zhang H, Roberts G, Kurukulaaratchy RJ, Arshad SH, Ewart S (2013) Effect of GSTM2–5 polymorphisms in relation to tobacco smoke exposures on lung function growth: A birth cohort study. BMC Pulm Med 13:56. 10.1186/1471-2466-13-56 24004509 10.1186/1471-2466-13-56 PMC3846453

[28] Sun C, Gu Y, Chen G, Du Y (2019) Bioinformatics analysis of stromal molecular signatures associated with breast and prostate cancer. J Comput Biol 26(10):1130–1139. 10.1089/cmb.2019.0045 31180245 10.1089/cmb.2019.0045

[29] Chen Y, Li B, Wang J, Liu J, Wang Z, Mao Y, Liu S, Liao X, Chen J (2020) Identification and verification of the prognostic value of the glutathione S-transferase Mu genes in gastric cancer. Oncol Lett 20(4):100. 10.3892/ol.2020.11961 32831919 10.3892/ol.2020.11961 PMC7439103

[30] Wang SC, Huang CC, Shen CH, Lin LC, Zhao PW, Chen SY, Deng YC, Liu YW (2016) Gene expression and DNA methylation status of glutathione S-transferase Mu1 and Mu5 in urothelial carcinoma. PLoS One 11(7):e0159102. 10.1371/journal.pone.0159102 27404495 10.1371/journal.pone.0159102 PMC4942074

[31] Peng DF, Razvi M, Chen H, Washington K, Roessner A, Schneider-Stock R, El-Rifai W (2009) DNA hypermethylation regulates the expression of members of the Mu-class glutathione S-transferases and glutathione peroxidases in Barrett’s adenocarcinoma. Gut 58(1):5–15. 10.1136/gut.2007.146290 18664505 10.1136/gut.2007.146290 PMC2845391

[32] Uppugunduri CRS, Muthukumaran J, Robin S, Santos-Silva T, Ansari M (2022) In silico and in vitro investigations on the protein-protein interactions of glutathione S-transferases with mitogen-activated protein kinase 8 and apoptosis signal-regulating kinase 1. J Biomol Struct Dyn 40(3):1430–1440. 10.1080/07391102.2020.1827036 32996404 10.1080/07391102.2020.1827036

[33] Yang S, Li F, Lu S, Ren L, Bian S, Liu M, Zhao D, Wang S, Wang J (2022) Ginseng root extract attenuates inflammation by inhibiting the MAPK/NF-kappaB signaling pathway and activating autophagy and p62-Nrf2-Keap1 signaling in vitro and in vivo. J Ethnopharmacol 283:114739. 10.1016/j.jep.2021.114739 34648903 10.1016/j.jep.2021.114739

[34] Shao W, Wang X, Liu Z, Song X, Wang F, Liu X, Yu Z (2023) Cyperotundone combined with adriamycin induces apoptosis in MCF-7 and MCF-7/ADR cancer cells by ROS generation and NRF2/ARE signaling pathway. Sci Rep 13(1):1384. 10.1038/s41598-022-26767-x 36697441 10.1038/s41598-022-26767-x PMC9877033

[35] Xu F, Xie Q, Li YW, Jing QQ, Liu XJ, Xu YC, Wang X, Liu L, Kim G, Choi Y, Guo Y, Zhang E, Jin CY (2022) Suppression of JNK/ERK dependent autophagy enhances Jaspine B derivative-induced gastric cancer cell death via attenuation of p62/Keap1/Nrf2 pathways. Toxicol Appl Pharmacol 438:115908. 10.1016/j.taap.2022.115908 35123989 10.1016/j.taap.2022.115908

[36] Sample A, Zhao B, Wu C, Qian S, Shi X, Aplin A, He YY (2018) The autophagy receptor adaptor p62 is up-regulated by UVA radiation in melanocytes and in melanoma cells. Photochem Photobiol 94(3):432–437. 10.1111/php.12809 28715145 10.1111/php.12809 PMC5771989

[37] Hwang SK, Jeong YJ, Chang YC (2020) PDCD4 inhibits lung tumorigenesis by the suppressing p62-Nrf2 signaling pathway and upregulating Keap1 expression. Am J Cancer Res 10(2):424–439 32195018 PMC7061761

[38] Li T, Jiang D, Wu K (2020) p62 promotes bladder cancer cell growth by activating KEAP1/NRF2-dependent antioxidative response. Cancer Sci 111(4):1156–1164. 10.1111/cas.14321 31967368 10.1111/cas.14321 PMC7156869

[39] Liu T, Sun L, Zhang Y, Wang Y, Zheng J (2022) Imbalanced GSH/ROS and sequential cell death. J Biochem Mol Toxicol 36(1):e22942. 10.1002/jbt.22942 34725879 10.1002/jbt.22942

[40] GilardiniMontani MS, Tarquini G, Santarelli R, Gonnella R, Romeo MA, Benedetti R, Arena A, Faggioni A, Cirone M (2022) p62/SQSTM1 promotes mitophagy and activates the NRF2-mediated antioxidant and anti-inflammatory response restraining EBV-driven B lymphocyte proliferation. Carcinogenesis 43(3):277–287. 10.1093/carcin/bgab116 34958370 10.1093/carcin/bgab116

[41] Su X, Guo W, Yuan B, Wang D, Liu L, Wu X, Zhang Y, Kong X, Lin N (2021) Artesunate attenuates bone erosion in rheumatoid arthritis by suppressing reactive oxygen species via activating p62/Nrf2 signaling. Biomed Pharmacother 137:111382. 10.1016/j.biopha.2021.111382 33761603 10.1016/j.biopha.2021.111382

[42] Lu Q, Gu W, Luo C, Wang L, Hua W, Sun Y, Tang L (2021) Phytochemical characterization and hepatoprotective effect of active fragment from Adhatoda vasica Nees. against tert-butyl hydroperoxide induced oxidative impairment via activating AMPK/p62/Nrf2 pathway. J Ethnopharmacol 266:113454. 10.1016/j.jep.2020.113454 10.1016/j.jep.2020.113454 33065254

[43] Liao W, Wang Z, Fu Z, Ma H, Jiang M, Xu A, Zhang W (2019) p62/SQSTM1 protects against cisplatin-induced oxidative stress in kidneys by mediating the cross talk between autophagy and the Keap1-Nrf2 signalling pathway. Free Radic Res 53(7):800–814. 10.1080/10715762.2019.1635251 31223046 10.1080/10715762.2019.1635251

[44] Yin Y, Zhu P, Luo T, Xia X (2020) Association of single-nucleotide polymorphisms in antioxidant genes and their gene-gene interactions with risk of male infertility in a Chinese population. Biomed Rep 13(1):49–54. 10.3892/br.2020.1306 32494361 10.3892/br.2020.1306 PMC7257938

[45] Zhang W, Gao J, Lu L, Bold T, Li X, Wang S, Chang Z, Chen J, Kong X, Zheng Y, Zhang M, Tang J (2021) Intracellular GSH/GST antioxidants system change as an earlier biomarker for toxicity evaluation of iron oxide nanoparticles. NanoImpact 23:100338. 10.1016/j.impact.2021.100338 35559839 10.1016/j.impact.2021.100338

[46] Zeng Q, Zhou T, Zhao F, Xiong D, He B, Hua Q, Lin M, Deng L, Sang X, Xie W, Chen J, Wang Z, Ren L, Luo Z, Huang X, Liu W, Tang S (2022) p62-Nrf2 regulatory loop mediates the anti-pulmonary fibrosis effect of bergenin. Antioxidants (Basel) 11(2). 10.3390/antiox11020307 10.3390/antiox11020307 PMC8868171 35204190

